# RETRACTION: Synergistic Antiproliferative Effects of Combined *γ*‐Tocotrienol and PPAR*γ* Antagonist Treatment Are Mediated through PPAR*γ*‐Independent Mechanisms in Breast Cancer Cells

**DOI:** 10.1155/ppar/9797132

**Published:** 2026-07-14

**Authors:** PPAR Research

**RETRACTION**: A. Malaviya, P. W. Sylvester, “Synergistic Antiproliferative Effects of Combined *γ*‐Tocotrienol and PPAR*γ* Antagonist Treatment Are Mediated through PPAR*γ*‐Independent Mechanisms in Breast Cancer Cells,” *PPAR Research*, 2014, 439146, https://doi.org/10.1155/2014/439146

The above article, published online on 04 March 2014 in Wiley Online Library (http://wileyonlinelibrary.com), has been retracted by agreement between the journal Associate Editor, Anjali Chauhan; and John Wiley & Sons Ltd. The retraction has been agreed due to concerns identified with the figures. Specifically:

• The following blots appear to be identical:
  ◦ *β*‐Actin blots in Figure 4b
  ◦ *β*‐Actin blots in Figure 5a
  ◦ 24 h *β*‐actin blots in Figure 12a (first 6 lanes)
  ◦ 48 h *β*‐actin blots in Figure 12a
  ◦ All *α*‐tubulin blots in Figure 11 a and 11b of the author’s previous work [1]
• The following blots appear to be identical:
  ◦ *β*‐Actin blots in Figure 7a
  ◦ Both sets of *β*‐actin blots in Figure 8a
  ◦ *β*‐Actin blots in Figures 9a and 9b
• The *β*‐actin blots in Figure 9d appear identical to the PPAR*γ*/*β*‐actin blots in Figure 8b
• The Scr.RNA/*β*‐actin blots in Figure 8b appear highly similar to the *β*‐actin blots in Figure 9c (lanes 2‐7)
• We have detected instances of potential undeclared splicing in the PGDS bands in Figure 9b
• The *β*‐actin bands in Figures 11a and 11b appear to be identical
• The 24 h bands shown in Figure 12a appear identical to the 24 h bands shown in Figure 11b of the author’s previous work [1]
• The 96 h bands shown in Figure 12a appear identical to the 96 h bands shown in Figure 11b of the author’s previous work [1]

After assessment of the concerns and the author’s response, the results and conclusions can no longer be considered reliable, and the article is therefore retracted.

P. W. Sylvester agrees to the retraction, whilst A. Malaviya was unresponsive.
